# Effect of Common Drinking Water Disinfectants, Chlorine and Heat, on Free *Legionella* and Amoebae-Associated *Legionella*

**DOI:** 10.1371/journal.pone.0134726

**Published:** 2015-08-04

**Authors:** Sílvia Cervero-Aragó, Sarah Rodríguez-Martínez, Antoni Puertas-Bennasar, Rosa M. Araujo

**Affiliations:** 1 Departament de Microbiologia, Facultat de Biologia, Universitat de Barcelona, Av. Diagonal 643, 08028, Barcelona, Spain; 2 Water Hygiene, Institute for Hygiene and Applied Immunology, Medical University of Vienna, Kinderspitalgasse 15, A-1090, Vienna, Austria; 3 Department of Biology and Environment, Faculty of Natural Sciences, University of Haifa, Oranim, 36006, Tivon, Israel; NERC Centre for Ecology & Hydrology, UNITED KINGDOM

## Abstract

Chlorine and thermal treatments are the most commonly used procedures to control and prevent *Legionella *proliferation in drinking water systems of large buildings. However, cases of legionellosis still occur in facilities with treated water. The purpose of this work was to model the effect of temperature and free chlorine applied in similar exposure conditions as in drinking water systems on five *Legionella* spp. strains and two amoebal strains of the genera *Acanthamoeba*. Inactivation models obtained were used to determine the effectiveness of the treatments applied which resulted more effective against *Legionella* than *Acanthamoeba*, especially those in cystic stages. Furthermore, to determine the influence of the relationship between *L*. *pneumophila* and *Acanthamoeba* spp. on the treatment effectiveness, inactivation models of the bacteria-associated amoeba were also constructed and compared to the models obtained for the free living bacteria state. The *Legionella*-amoeba association did not change the inactivation models, but it reduced the effectiveness of the treatments applied. Remarkably, at the lowest free chlorine concentration, 0.5 mg L^-1^, as well as at the lowest temperatures, 50°C and 55°C, the influence of the *Legionella*-amoeba associate state was the strongest in reducing the effectiveness of the treatments compared to the free *Legionella* state. Therefore, the association established between *L*. *pneumophila* and amoebae in the water systems indicate an increased health risk in proximal areas of the system (close to the tap) where lower free chlorine concentrations and lower temperatures are commonly observed.

## Introduction

Maintaining a high-quality drinking water in distribution systems is one of the goals of health authorities in many countries. Thus, several national standards have been established to provide high water quality, including disinfection techniques to control and prevent *Legionella* colonization [1–3]. However, when insufficiently applied, the survival of bacteria can promote a rapid re-colonization of the system [4–6]. This applies in particular to domestic hot water systems, which represent a source of human infections by *Legionellae* [7,8]. Two of the most common disinfection techniques used worldwide against *Legionella* are chlorination and thermal treatments. Free chlorine is mostly used at a low concentration (0.2–0.5 mg L^-1^) as a secondary disinfectant for the maintenance of water quality in distribution systems [3,9] or at higher concentrations as an installation disinfection treatment called hyperchlorination. This process is usually effective just for short periods of time [10,11]. In the case of thermal treatments, as suggested by the World Health Organization (WHO) and recommended in the Spanish guidelines, water flow temperature is kept at a minimum of 60°C when leaving the heating unit and at least 50°C when it reaches the tap [2,12]. However, even these temperatures have been shown to be insufficient to control *Legionella* proliferation in the hot water systems of several buildings [13,14].

The association established between the different microorganisms in the water systems are directly related to the effectiveness of the disinfection treatments applied [4,15]. Free-living amoebae (FLA) are eukaryotic microorganisms, commonly found in drinking water systems, and phagocyte bacteria, their nutritional source. However, some bacterial genera have developed strategies to survive the grazer effect of amoebae [16]. In particular, Rowbotham described for the first time in 1980 that *L*. *pneumophila* not only survive digestion by amoeba but also use the amoebae host nutritional sources to replicate intracellularly [17]. This intracellular state also protects *Legionella* against environmental factors and water disinfection treatments [5,18–20].

The aim of this work was to model the effect of free chlorine and temperature commonly used in building water systems on both *Legionella* and its amoeba hosts *Acanthamoeba*. For this study, five *Legionella* strains (including *L*. *pneumophila* and *L*. *longbeachae*) and two *Acanthamoeba* strains were selected. The two *Acanthamoeba* life stages, trophozoites and cysts, were treated separately. In addition, the influence of the association between *Legionella* and *Acanthamoeba* on the effectiveness of the treatments applied was determined by comparing the estimated models for the two *Legionella* states, free or in association with amoebae.

Bacterial and amoebal strains were chosen according to their previously reported pathogenicity. Whereas most legionellosis cases in Europe and the USA have been attributed to *L*. *pneumophila*, especially to serogroup 1, the causal agent of legionellosis in Australia and New Zealand was *L*. *longbeachae* [21,22]. On the other hand, *Acanthamoeba* genera are one of the most prevalent FLAs in drinking water systems and are the causative agent of an increasing number of *Acanthamoeba* keratitis [23]. Finally, although the data reported in the current work were obtained from *in vitro* experiments, the fact that the same quantification methods and analyses were used to study the effectiveness of the two most common disinfection treatments enables a direct comparison between them. That knowledge is essential in preventing *Legionella* infections.

## Material and Methods

### Strains of *Legionellae* and amoeba

Inactivation studies were conducted with five *Legionella* strains. Three reference strains were obtained from the American Type Culture Collection (ATCC): *Legionella pneumophila* serogroup 1 ATCC 33152, *Legionella pneumophila* serogroup 7 ATCC 33823 and *Legionella longbeachae* ATCC 33462. Two environmental strains were previously isolated from Catalonian hot tap water: *Legionella pneumophila* serogroup 1 and *Legionella pneumophila* serogroup 8 [14]. The five strains were stored at –80°C in Ringer 1/40 (prepared by diluting Ringer ¼ solution (Scharlau) ten-fold) with 15% glycerol (Panreac).

The inactivation of FLA was performed using 2 different strains: a reference strain obtained from the Culture Collection of Algae and Protozoa (CCAP), *Acanthamoeba castellanii* CCAP 1534/2, and an environmental strain previously isolated from Catalonian hot tap water, *Acanthamoeba* sp. 155 [24]. The two strains were stored in the cystic stage at –80°C in Ringer 1/40 (Scharlau) with 20% glycerol.

### Preparation of test suspensions and viability quantification after treatments

#### Legionella spp. Suspensions

*Legionella* strains were cultured on BCYE (buffered charcoal-yeast extract) supplemented with GVPC (MAIM, Spain) at 37°C for 72 h. Suspensions were prepared as described in a previous study [25]. The concentrations of the tested *Legionella* suspensions were approximately 5 × 10^5^ cfu/mL. The treatments were applied as explained in the “Free chlorine treatments” and “Thermal disinfection treatments” sections below.

#### Legionella spp. quantification after treatments

After each of the disinfection treatments, ten-fold serial dilutions were made in Ringer 1/40 for each sample and transferred to BCYE plates for enumeration of *Legionella* culturable colony-forming units. The plates were incubated at 37°C for up to 10 days. In the case of free chlorine treated samples, 100 μL of sodium thiosulfate 3% (Panreac) were added before plating the samples to neutralize the remaining chlorine at the different experimental times.

#### FLA suspensions

For each of the two amoeba strains, *A*. *castellanii* CCAP 1534/2 and *Acanthamoeba* sp. 155, two tests were performed according to their life stage: trophozoite or cyst. For the *Acanthamoeba* trophozoite experiments, axenic cultures were obtained and maintained by sub-culturing them in PYG (proteose-peptone-yeast extract-glucose) medium (ATCC 712) in 25 cm^3^ Roux flasks as previously described [24]. After the trophozoites were grown to confluence for 2–3 days at 30°C, they were recovered from the tissue culture flasks with a soft shake. *Acanthamoeba* cysts were obtained by culturing amoebal strains on non-nutrient agar (NNA) plates seeded with fresh *Escherichia coli* at 30°C for 10±2 days. At that time, cultures composed of more than 90% of double-walled cysts were harvested with Ringer 1/40 [24]. Finally, trophozoite and cyst suspensions obtained from the 2 FLA strains were centrifuged at 800 x g for 15 min, resuspended in Ringer 1/40, and then adjusted to a final concentration of 1 x 10^5^ amoeba cells/mL using a Neubauer chamber. The viability of the initial suspensions was quantified following the Most Probable Number (MPN) method previously described [24]. The MPN values were obtained from MPN tables [26]. The initial suspensions were considered time 0.

#### FLA viability quantification after treatments

Once the thermal and chlorination treatments were performed, quantification of trophozoites and cysts was performed using MPN [24]. In the case of free chlorine treated samples, 100 μL of sodium thiosulfate 3% (Panreac) were added before plating the samples to neutralize the remaining chlorine.

#### Co-culture of L. pneumophila and Acanthamoeba strains

The *L*. *pneumophila* sg. 1 environmental strain was co-cultured with either *A*. *castellanii* CCAP 1534/2 or *Acanthamoeba* sp. 155 as follows. Trophozoites of *Acanthamoeba* strains were grown at 30°C for two days in 75 cm^3^ tissue culture flasks containing 30 mL of PYG medium. The *L*. *pneumophila* sg. 1 environmental strain was cultured on BCYE agar plates supplemented with GVPC (MAIM, Spain) at 37°C for 72 h. *Acanthamoeba* trophozoites were adjusted to a concentration of 1 × 10^5^ trophozoites/mL, as explained in FLA suspensions section, in a suspension of PYG medium to a final volume of 15 mL and incubated at 30°C for 30 min to promote cell attachment. After that, a suspension of 5 × 10^7^ *Legionella* cells/mL, obtained as explained in *Legionella* suspensions section, was added to the same tissue flask at a multiplicity of infection (MOI) of 100:1. Flasks were incubated at 30°C for 90 min. Then, four washing steps, soft shaking and replacing the supernatant with fresh PYG medium were performed to wash out the remaining extracellular bacteria. The efficacy of the washing procedure was confirmed by microscopic observation. Finally, trophozoites containing *Legionella* cells were resuspended in 30 mL of fresh PYG medium and incubated at 30°C for 40 h or 48 h. To perform inactivation experiments, co-culture suspensions were recovered from the tissue culture flasks with a soft shake, centrifuged at 800 x g for 15 min and resuspended in Ringer 1/40 in order to recover all amoebae cells and eliminate possible extracellular bacteria and other cellular debris.

#### L. pneumophila co-culture monitoring

The intracellular presence of the *L*. *pneumophila* sg. 1 environmental strain within an *Acanthamoeba* strain was monitored using fluorescence *in situ* hybridization (FISH). The suspensions were analyzed at different times after co-culture to determine the maximum number of *Legionella* cells within an amoeba cell. Briefly, 1 mL of the co-culture sample was washed twice by centrifugation at 1000 x g for 5 min adding fresh phosphate-buffered saline (PBS). Then, 900 μL of the supernatant were discarded and the pellet was resuspended in the remaining PBS. From there, 10 μL were placed on a 10-well Teflon slide (Medco Health Solutions, Inc., Germany). Slides were incubated at 30°C for 30 min to let the cells attach to the slide surface. The fixation of the samples was realized by incubating them for 10 min at room temperature in 20 μL of 4% paraformaldehyde (v/v PBS), washed once with PBS, and dehydrated, for 3 min, in an aqueous ethanol series (50, 80, and 96%). Fixed samples were then hybridized in 10 μL of hybridization buffer (25% [vol/vol] formamide, 0.9 M NaCl, 0.01% sodium dodecyl sulfate, 20 mM Tris-HCl [pH 8]) per well plus 1 μL (50 ng) of the LEGPNE1 FITC probe for the detection of the 16S rRNA of *L*. *pneumophila* [27] and 1 μL (50 ng) of the EUK516 probe specific for the 18S rRNA of the *Eukaria* Domain [28]. Slides were incubated at 46°C for 2 h in a humid chamber. The unbounded probes were then washed by incubating the slide in 50 mL of washing buffer (20 mM Tris-HCl [pH 8], 0.01% sodium dodecyl sulfate, 5 mM EDTA, 160 mM NaCl) at 48°C for 15 min. Slides were then rinsed in ice-cold 96% ethanol, air dried in the dark and mounted in Citifluor (Citifluor, Ltd. London, United Kingdom). The preparations were observed by a Nikon Eclipse 8000 epifluorescence microscope and photographs were processed with the software NIS Elements BR 2.3 (Nikon). In parallel, co-culture samples from these time points were plated on BCYE and NNA to quantify *Legionella* and amoeba replication under co-culture conditions. A control of axenic *Legionella* growth on PYG media was also analyzed.

#### Quantification of the inactivation of L. pneumophila associated with Acanthamoeba strains

After chlorine and thermal treatments, a ten-fold serial dilution in Ringer 1/40 of each co-culture sample was transferred to BCYE plates for enumeration of *L*. *pneumophila* sg. 1 viable colony forming units, as described above. *L*. *pneumophila* counts were compared with the results obtained from plating an untreated suspension.

### Free chlorine disinfection treatments

#### Waters tested

All disinfection experiments were performed in commercial natural mineral water at room temperature. The pH of the water was 7.2.

#### Material used for free chlorine treatments

Glassware material was prepared as described previously [29]. Briefly, glassware was soaked overnight in a solution of at least 100 mg free chlorine L^-1^. Flasks were then rinsed with chlorine-demand-free water (BDF) and muffled for 4 h at 400°C. After each experiment, glassware was soaked in free chlorine and rinsed in demand free water. BDF water was prepared as described by [29]. Free chlorine concentration was measured using the *N*,*N*-diethyl-phenylenediamine (DPD) method (BOE 140:2003) by using HI 95711 Free & Total Chlorine (Hanna Instruments). A chlorine stock solution of 100 mg L^-1^ was prepared using bleach (a commercial sodium hypochlorite solution of approximately 42 g of free chlorine L^-1^) intended for tap water disinfection. The solution was kept at 4°C for a maximum of one month. The stock solution was diluted to achieve free chlorine concentrations used in the disinfection experiments.

#### Experimental protocol

One hundred milliliters of natural mineral water were placed in chlorine-demand-free glass flasks. First, to determine the natural free-chlorine decay in the water matrix, 1 mg L^-1^ of free-chlorine was added to the water flask and immediately stirred. The flask was sampled to determine the initial (at 10 s) free-chlorine concentration in the absence of any chlorine demand and sampled again every 15 min for 1 h. Next, a second flask with 100 mL of mineral water was inoculated with 100 μL of microorganism suspensions to determine the effect of mineral water on the different microorganisms. Finally for microorganism inactivation, 100 mL of mineral water was inoculated with the free-chlorine stock solution and immediately stirred. The flask was sampled to check that water reached the chosen chlorine concentration. After that, 100 μL of a microorganism suspension was inoculated into the flask. At the chosen times, 2 mL samples were successively transferred into 10 mL collection tubes containing 100 μL of a sterile 3% sodium thiosulfate solution to quench the residual free chlorine. Several chlorine concentrations were tested: 0.2 mg L^-1^ and 0.5 mg L^-1^ in *Legionella* experiments, 1.2 mg L^-1^ and 1.5 mg L^-1^ in *Acanthamoeba* experiments and 0.5 mg L^-1^, 1.2 mg L^-1^ and 2.5 mg L^-1^ in co-culture experiments.

### Thermal disinfection treatments

To study the inactivation of microorganisms by thermal treatment, a microcosm system was designed using dialysis bags (Medicell International Ltd., London, UK) [24]. Briefly, dialysis bags containing 2 mL of each microorganism suspension were sealed with a knot and placed in a water bath (Water Bath 1002–1013, GFL). Five experimental temperatures were tested, 50, 55, 60, 65 and 70°C, for various exposure times.

### Statistical analysis

The inactivation of different microorganisms was defined as a logarithmic reduction (N/N_0_), where N_0_ and N were the concentration of cultivable organisms of *Legionella* or the MPN of the amoebae before and after inactivation treatments, respectively. The data reported in this study were obtained from independent triplicates.

The results are reported as means ± standard deviation (SD). Experimental conditions were statistically analyzed using one-way ANOVA tests (Statgraphics Plus 5.1, Rockville, MD, USA); *p* values less than 0.05 were considered statistically significant. After the ANOVA test, the pairwise Fisher’s LSD (Least Significant Difference) test was used to discern between means in cases of significant difference (Statgraphics Plus 5.1). Significantly different means were plotted by a different letter on the bars, whereas bars with the same letter indicate no significant differences. Before any statistical analysis, the data was checked for compliance with ANOVA assumptions. The graphs were plotted using GraphPad Prism 4 (GraphPad Software, San Diego, CA, USA).

Inactivation kinetics that describe how *Legionella* and FLA strains behaved over the exposure time to the disinfectants used was modeled by a first-order model characterized by a constant decay rate and a two phase decay model characterized by a two different decay rates. The correlation coefficient R^2^ was used to check the robustness of the chosen models. In addition, the required treatments for 3 log and 4 log reductions were calculated. These parameters are described as the time (min) required to reduce the cultivability of the initial microbial population by 99.9% or 99.99%, and it corresponds to the value of X in the equation when Y = 3 or when Y = 4. All models were fit to the experimental data using the program GraphPad Prism 4.

## Results

### Free chlorine inactivation of *Legionella* spp. strains

**Fig 1:**
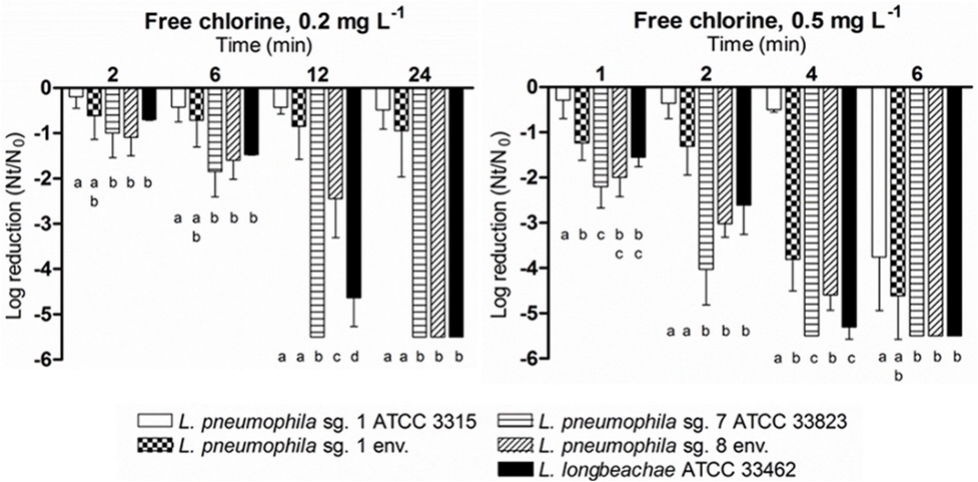
Effect of free chlorine 0.2 mg L^-1^ and 0.5 mg L^-1^, on the inactivation of 5 *Legionella* strains. Bacterial inactivation was determined using viable counts on BCYE agar medium. Data are presented as means ± SD (columns and error bars; n = 3). Statistical differences between means within each time point were represented assigning different letters to the bar plot. The same letter was assigned to bars with no significant differences between them. Statistical analyses were performed by ANOVA and pairwise Fisher’s LSD test (*p*<0.05).

Five strains of *Legionella* spp., three from culture collections and two environmental isolates, were exposed to two free chlorine concentrations typically found in drinking water (Fig 1). Previous controls performed on the water matrix showed that free chlorine concentration remained stable for the experimental times chosen in the absence of microorganisms, as did the concentrations of the microorganisms in absence of chlorine. At 0.2 mg L^-1^ and 0.5 mg L^-1^, *L*. *pneumophila* sg. 1 ATCC 33152 was the most resistant strain (p<0.05). Furthermore, results showed significant differences (*p*<0.0001) between the inactivation of the two *L*. *pneumophila* sg. 1 strains compared to the three other strains studied when exposed at 0.2 mg L^-1^. *L*. *pneumophila* sg.7 ATCC 33823, *L*. *pneumophila* sg. 8 and *L*. *longbeachae* ATCC 33462 reached a 5-log reduction in cultivability after 24 min of treatment, whereas the *L*. *pneumophila* sg. 1 strain was approximately reduced by 1 log (Fig 1). At 0.5 mg L^-1^, significant differences were also observed between *L*. *pneumophila* sg. 1 and non-sg. 1 strains (*p*<0.05, *p*<0.001). After 4 min of treatment, cultivability in 4 out of the 5 *Legionella* strains was reduced by nearly 4 logs. *L*. *pneumophila* sg. 1 ATCC 33152 was the only exception.

The inactivation kinetics of *Legionella* strains differed depending on the chlorine concentration to which they were exposed (Table 1). At 0.2 mg L^-1^, inactivation of the *L*. *pneumophila* sg. 1 strains fit a biphasic decay model characterized by an initial decay followed by a slightly steep slope, whereas the inactivation of the other 3 strains fit a first-order model represented by a straight line. However, at 0.5 mg L^-1^, the inactivation of all *Legionella* strains fit a first-order model. At 0.2 mg L^-1^, due to their biphasic decay model, none of the *L*. *pneumophila* sg. 1 strains reached a 4-log reduction. However, *L*. *pneumophila* sg. 7 ATCC 33823, *L*. *pneumophila* sg. 8 and *L*. *longbeachae* ATCC 33462 did reach a 4-log reduction after a contact time of 9, 20 and 11 min, respectively. At 0.5 mg L^-1^ of free chlorine, the cultivability of the non-*L*. *pneumophila* sg. 1 strains was reduced 4 logs after 2 to 3 min, whereas *L*. *pneumophila* sg. 1 strains required 4 to 8 min.

**Table 1:** Calculated time for a 4-log reduction of five *Legionella* strains at 0.2 mg L^-1^ and 0.5 mg L^-1^ of free chlorine. Inactivation kinetics adjusted to a biphasic decay model (*) and to a first-order (straight line) model. R^2^ values showed the robustness of the model. **NA** (not achieved).

*Legionella* strains	Calculated time (min) to reduce 4 logs			
	0.2 mg L^-1^	R^2^	0.5 mg L^-1^	R^2^
*L*. *pneumophila* sg. 1 ATCC 33152	NA*	0.99	8	0.72
*L*. *pneumophila* sg. 1 env.	NA*	0.96	4	0.95
*L*. *pneumophila* sg. 7 ATCC 33823	9	0.95	2	0.95
*L*. *pneumophila* sg. 8 env.	20	0.91	3	0.95
*L*. *longbeachae* ATCC 33462	11	0.96	3	0.99

### Free chlorine inactivation of *Acanthamoeba* strains

**Fig 2:**
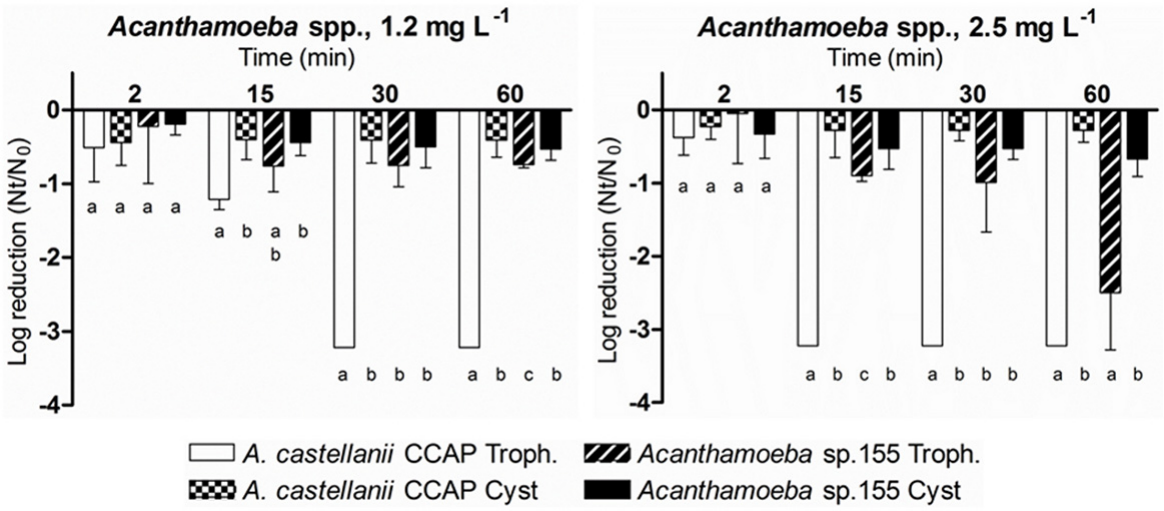
Effect of free chlorine, 1.2 mg L^-1^ and 2.5 mg L^-1^, on the inactivation of 2 *Acanthamoeba* strains treating separately trophozoites and cysts. Amoebal inactivation was determined using an adaptation of the MPN method. Data are presented as means ± SD (columns and error bars; n = 3). Statistical differences between means within each time point were represented assigning different letters to the bar plot. The same letter was assigned to bars with no significant differences between them. Statistical analyses were performed by ANOVA and pairwise Fisher’s LSD test (*p*<0.05).

To investigate the effectiveness of free chlorine exposure against FLA, two strains were tested: *A*. *castellanii* CCAP 1534/2 and the environmental isolate *Acanthamoeba* sp. 155. Each experiment was performed considering the amoeba life stages: trophozoite or cyst. Because *Acanthamoeba* was found to be resistant to the typical free chlorine concentrations found in drinking water (data not shown), trophozoites and cysts were exposed to 1.2 mg L^-1^ and 2.5 mg L^-1^ (Fig 2).

The results showed that the efficacy of chlorine was significantly higher on trophozoites compared to cysts, particularly at 2.5 mg L^-1^ (*p*<0.05). *A*. *castellanii* CCAP 1534/2; trophozoites were significantly more sensitive to chlorine being reduced approximately 3 logs after a contact time of 30 min at 1.2 mg L^-1^ (*p*<0.001) and after 15 min at 2.5 mg L^-1^ (*p*<0.05, *p*<0.001). The cultivability of *Acanthamoeba* sp. 155 trophozoites was reduced less than 3 log in all of the conditions tested. In the case of the cysts, chlorine treatments at 1.2 mg L^-1^ or at 2.5 mg L^-1^ reduced cultivability by less than one log for both *Acanthamoeba* strains tested.

The inactivation kinetics for the two FLA strains fit a biphasic decay model (Table 2). Trophozoites of *A*. *castellanii* CCAP 1534/2 experienced a 3-log reduction in cultivability after a contact time of 29 min at 1.2 mg L^-1^ or after 14 min at 2.5 mg L^-1^, whereas the cultivability of *Acanthamoeba* sp. 155 trophozoites was reduced 3 logs after 74 min at 2.5 mg L^-1^. The inactivation models of *Acanthamoeba* cysts could not be calculated due to the high resistance observed at the chosen free chlorine concentrations (Table 2).

**Table 2:** Calculated time for a 3-log reduction of two *Acanthamoeba* strains treated separately by its life stage, cyst or trophozoite at 1.2 mg L^-1^ and 2.5 mg L^-1^ of free chlorine. Inactivation kinetics of amoeba strains adjusted to a first-order model and to a biphasic decay model (*). R^2^ values showed the robustness of the model. **NA** (not achieved).

*Acanthamoeba* strains	Life form	Calculated time (min) to reduce 3 logs			
		1.2 mg L^-1^	R^2^	2.5 mg L^-1^	R^2^
*A*. *castellanii* CCAP 1534/2	Trophozoite	29	0.96	14	0.99
	Cyst	NA*	0.99	NA*	0.99
*Acanthamoeba sp*. *155*	Trophozoite	NA*	0.99	74	0.97
	Cyst	NA*	0.99	NA*	0.95

### Thermal inactivation of *Legionella* spp. strains

**Fig 3:**
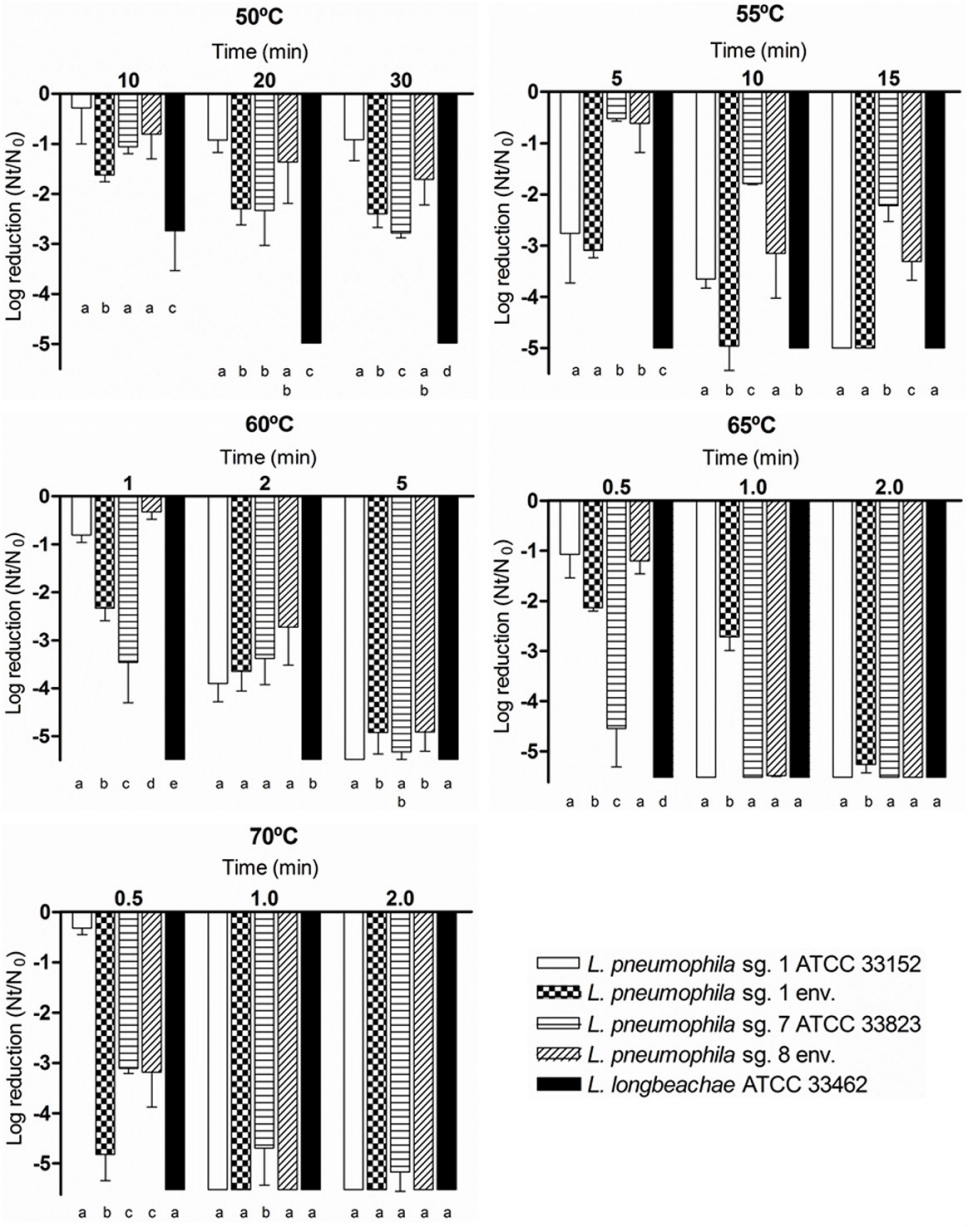
Effect of thermal treatments on the inactivation of 5 *Legionella* strains. Bacterial inactivation was determined using viable counts on BCYE agar medium. Data are presented as means ± SD (columns and error bars; n = 3). Statistical differences between means within each time point were represented assigning different letters to the bar plot. The same letter was assigned to bars with no significant differences between them. Statistical analyses were performed by ANOVA and pairwise Fisher’s LSD test (*p*<0.05).

A thermal treatment at five different temperatures, 50°C, 55°C, 60°C, 65°C and 70°C, was applied to the five *Legionella* spp. strains. The results showed significant differences between the inactivation patterns of the five strains, especially *L*. *longbeachae* ATCC 33462, which was significantly (*p*<0.001) the most sensitive to all the thermal treatments applied. Significant differences (*p*<0.05) were found among the rest of the strains used, but the behavior of each strain varied depending of the thermal treatment applied (Fig 3).

At 50°C, *L*. *longbeachae* ATCC 33462 was the most sensitive strain, reaching a 5-log inactivation after a 20 min exposure. *L*. *pneumophila* sg. 1 environmental and *L*. *pneumophila* sg. 7 ATCC 33823 showed a similar behavior at 50°C and were significantly more sensitive (p<0.05) to this temperature than *L*. *pneumophila* sg. 1 ATCC 33152 and *L*. *pneumophila* sg. 8. At 55°C, *L*. *longbeachae* ATCC 33462 reached a 5-log inactivation after 5 min. At that temperature, *L*. *pneumophila* sg. 1 ATCC 33152 and *L*. *pneumophila* sg. 1 environmental were significantly more sensitive (p<0.05) than the non-serogroup 1 strains. At 60°C, 65°C and 70°C, *L*. *pneumophila* strains had a similar behavior, except at the lowest exposure times, 0.5 and 1 min, where significant differences were found (*p*<0.05) (Fig 3).

The inactivation kinetics of the five *Legionella* strains fit a first-order model (straight line), shown in Table 3. For each strain and temperature tested, the required time to achieve a 4-log reduction was calculated. At 50°C, the most resistant strain (*p*<0.05) was *L*. *pneumophila* sg. 1 ATCC 33152, which required 117 min. The time needed to obtain a 4-log reduction for the other *L*. *pneumophila* strains ranged from 40–68 min. *L*. *longbeachae* ATCC 33462 was the most sensitive (*p*<0.001) strain at all the temperatures tested, reaching a 4-log reduction at 50°C in only 15 min. At 55°C, whereas *L*. *pneumophila* sg. 1 strains needed approximately 8–10 min to achieve a 4-log reduction, *L*. *pneumophila* sg. 8 and *L*. *pneumophila* sg. 7 ATCC 33823 needed between 16 and 25 min, respectively, to obtain the same 4-log reduction. At 60°C, 65°C and 70°C, the comparison of the calculated inactivation parameters showed narrow ranges as the temperature increased. *L*. *pneumophila* strains reached a 4-log reduction in a range between 2–4 min at 60°C and 1 min at 65°C and 70°C.

**Table 3:** Calculated time for a 4-log reduction of five *Legionella* strains at five different temperatures. Inactivation kinetics adjusted to first-order models (straight line). R^2^ values showed the robustness of the models.

***Legionella* strains**	**Calculated time (min) to reduce 4 logs**									
	**50°C**	R^2^	**55°C**	R^2^	**60°C**	R^2^	**65°C**	R^2^	**70°C**	R^2^
*L*. *pneumophila* sg. 1 ATCC 33152	117	0.8	10	0.92	2	0.90	0.8	0.88	0.9	0.79
*L*. *pneumophila* sg. 1 env.	46	0.84	8	0.98	3	0.83	1.4	0.90	0.6	0.82
*L*. *pneumophila* sg. 7 ATCC 33823	40	0.97	25	0.96	3	0.76	0.6	0.87	1.2	0.77
*L*. *pneumophila* sg. 8 env.	68	0.97	16	0.89	4	0.94	0.8	0.90	0.7	0.99
*L*. *longbeachae* ATCC 33462	15	0.94	2	0.88	—-	—-	—-	—-	—-	—-

### *L*. *pneumophila* growth associated with *Acanthamoeba* strains

**Fig 4:**
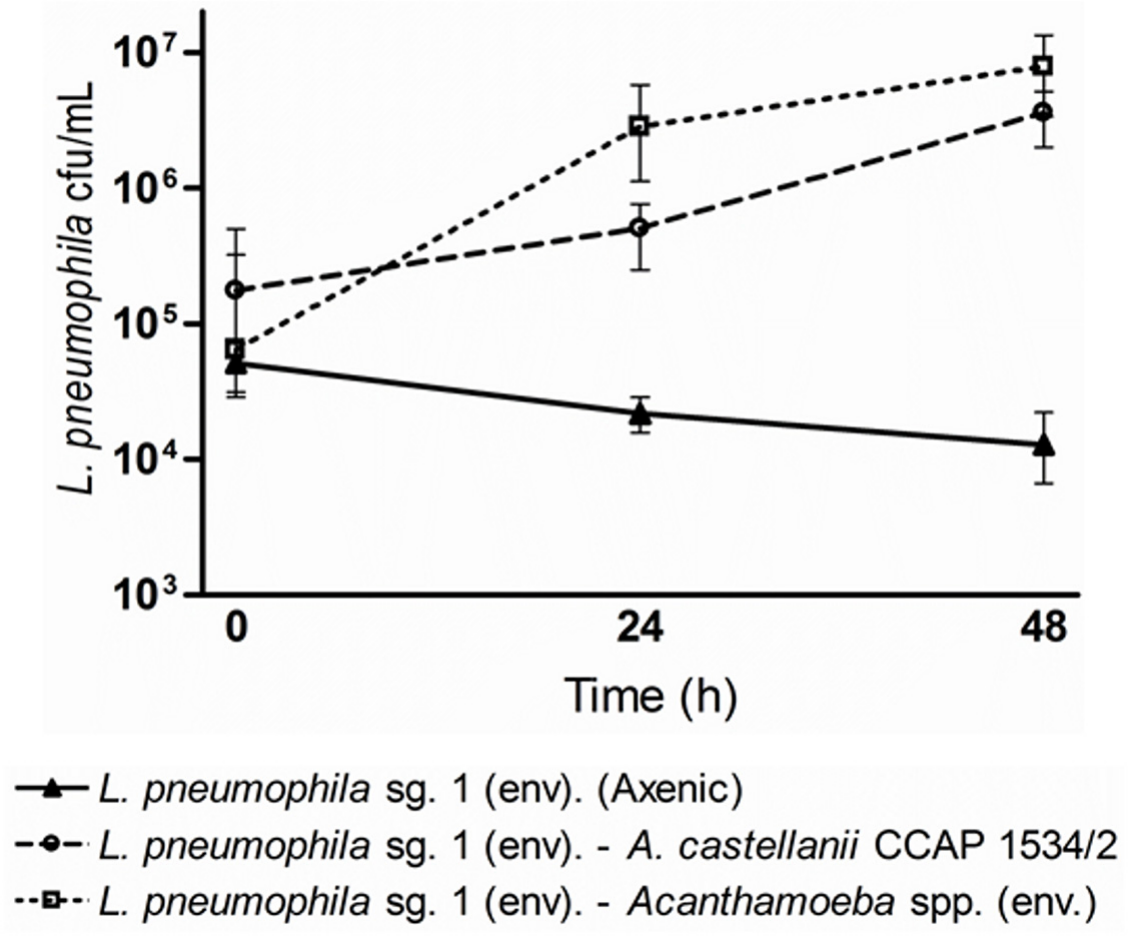
*L*. *pneumophila* sg. 1 growth in axenic conditions and in co-culture with the two *Acanthamoeba* strains in PYG liquid media. Samples were taken at different time points and plated on BCYE agar plates. Data are presented as means ± SD (error bars; n = 3).

**Fig 5:**
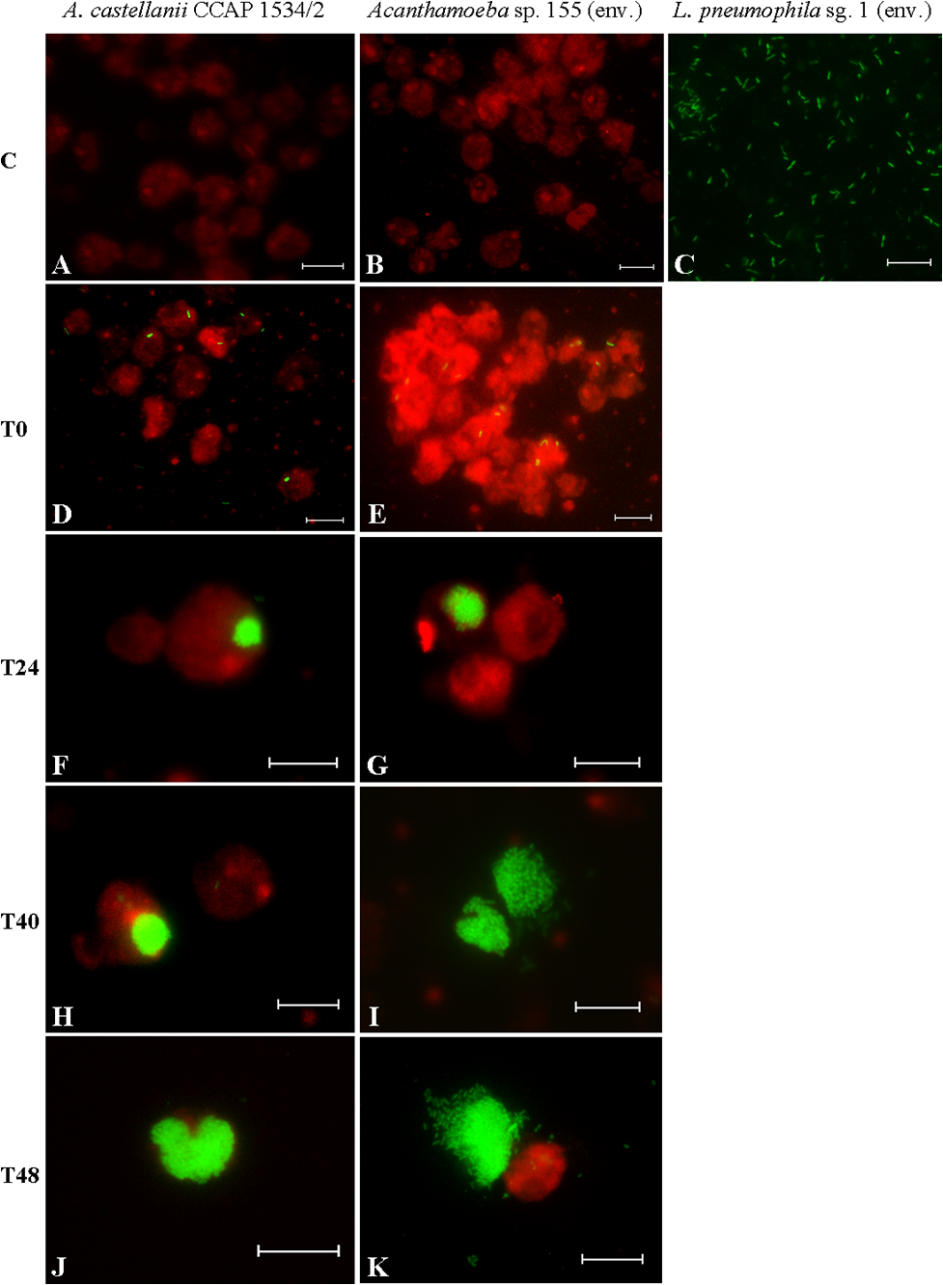
Pictures obtained by FISH using an epifluorescence microscope to monitor the intracellular presence of *L*. *pneumophila* sg. 1 (env.) within the two amoeba strains, *A*. *castellanii* CCAP 1534/2 (first column) and *Acanthamoeba* sp. 155 (env.) (second column). Negative controls of pure cultures are shown in the first line (**A**, **B** and **C**). Then, the presence of *L*. *pneumophila* was analysed at different time points: just after the co-culture protocol (**T0**) and after 24 h, 40 h and 48 h (**T24**, **T40** and **T48**, respectively). All samples, including the controls, were simultaneously hybridized with the LEGPNE1 probe (FITC, green) and the probe EUK 516 (Cy3, red). Pictures were taken at 1000X magnification, bar scale = 9.2 µm.

Two co-cultures of the *L*. *pneumophila* sg. 1 environmental strain with *A*. *castellanii* CCAP 1534/2 and with *Acanthamoeba* sp. 155 were tested. The results obtained from plating the co-cultures on BCYE agar showed that the bacteria had a different (*p*<0.05) replication rate depending on the amoeba strain (Fig 4). Whereas bacterial loads increased 1.3 logs after 48 h within *A*. *castellanii* CCAP 1534/2, bacterial loads increased 2 logs within *Acanthamoeba* sp. 155. In parallel, a negative control showed that *L*. *pneumophila* sg. 1 environmental did not grow on PYG media without the presence of amoebae. On the other hand, the results obtained from plating the co-cultures on NNA showed that amoebae populations were reduced a mean of 0.70 log after 48 h in the case of the *A*. *castellanii* CCAP co-culture and 0.64 logs after 40 h in the case of the *Acanthamoeba* sp. 155 co-culture. The intracellular presence of *L*. *pneumophila* was also monitored by fluorescence *in situ* hybridization (FISH) (Fig 5). All amoeba strains included in the study were tested before the co-culture experiments to confirm the absence of other intracellular *L*. *pneumophila* strains. FISH results showed that, for every time point, the number of trophozoites containing intracellular bacteria and the number of bacteria within each trophozoite was highly variable (Fig 5). That observation suggests that the replication rhythm of *L*. *pneumophila* within each *Acanthamoeba* trophozoite is not synchronized. The most appropriate time point for chlorine and temperature treatments was chosen according to the maximum number of *L*. *pneumophila* replicating vesicles observed. For *A*. *castellanii* CCAP 1534/2, this time was after 48 h of co-culture, whereas for *Acanthamoeba* sp. 155, it was after 40 h of co-culture.

### Effect of the association between *L*. *pneumophila* with *Acanthamoeba* strains on the effectiveness of the inactivation treatments applied

#### Free chlorine treatments

**Fig 6:**
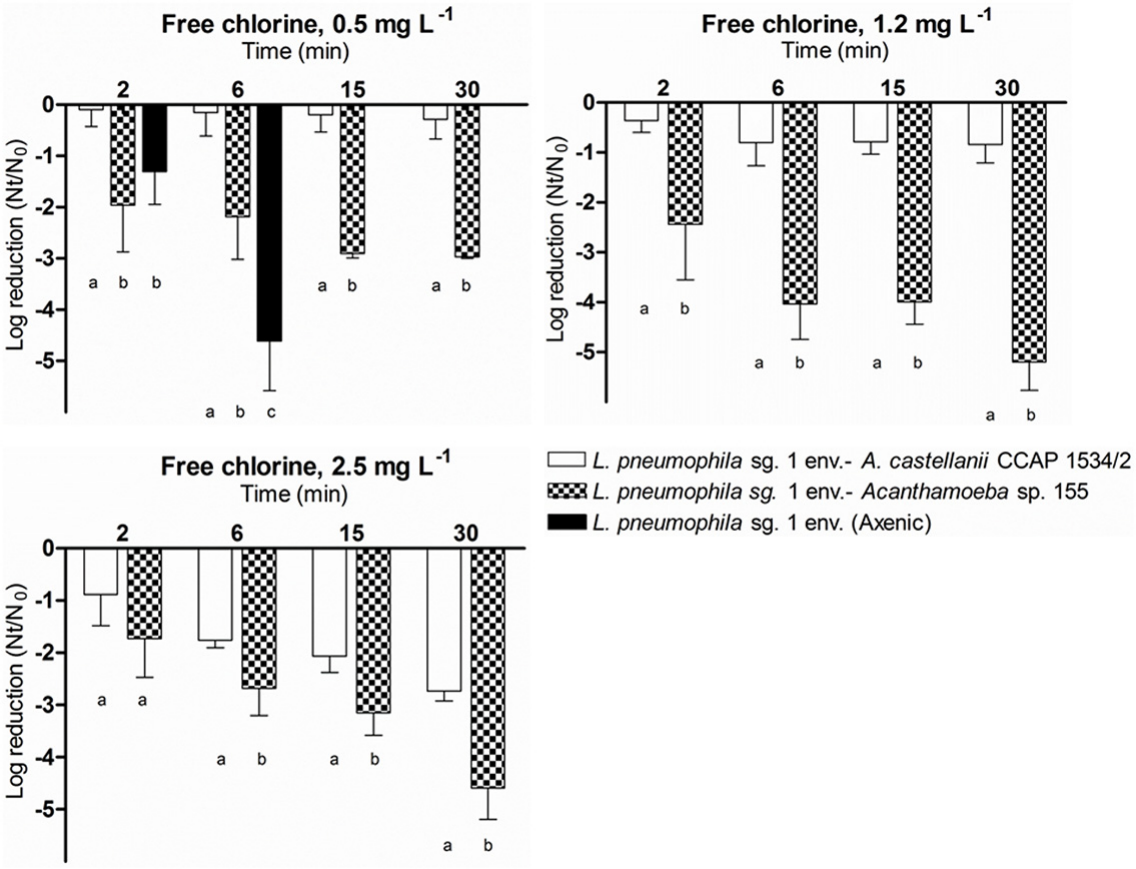
Effect of free chlorine, on the inactivation of *L*. *pneumophila* sg. 1 env. associated to two *Acanthamoeba* strains, *A*.*castellanii* CCAP 1534/2 and *Acanthamoeba* sp. 155 strains. Bacterial inactivation was determined using viable counts on BCYE medium. Data are presented as means ± SD (columns and error bars; n = 3). Statistical differences between means within each time point were represented assigning different letters to the bar plot. The same letter was assigned to bars with no significant differences between them. Statistical analyses were performed by ANOVA and pairwise Fisher’s LSD test (*p*<0.05).

The effect of free chlorine was evaluated on the *L*. *pneumophila* sg. 1 environmental strain in association with *A*. *castellanii* CCAP 1534/2 and *Acanthamoeba* sp. 155 (Fig 6). Significant differences were found between the inactivation of both associations tested, with *L*. *pneumophila* associated with *Acanthamoeba* sp. 155 being more sensitive to chlorine exposure than *L*. *pneumophila* associated with *A*. *castellanii* CCAP 1534/2.

At 0.5 mg L^-1^, significant differences (*p*<0.001) were found between the two associations tested. After 30 minutes of treatment, *L*. *pneumophila* associated with *Acanthamoeba* sp. 155 reached a 3-log reduction, whereas the cultivability of *L*. *pneumophila* associated with *A*. *castellanii* CCAP 1534/2 was less than one-log reduced. The results of the *L*. *pneumophila* sg. 1 environmental grown in axenic conditions is included in the graph to enable the comparison of their inactivation patterns. The results show significant differences (p<0.001) between the inactivation of the axenic *L*. *pneumophila* and *L*. *pneumophila* associated with protozoa. Axenic *L*. *pneumophila* reached a 5-log reduction after 6 min at 0.5 mg L^-1^, whereas *L*. *pneumophila* associated with *Acanthamoeba* sp. 155 was reduced 2 logs and *L*. *pneumophila* associated with *A*. *castellanii* CCAP 1534/2 was reduced less than one log.

At 1.2 mg L^-1^ and at 2.5 mg L^-1^, large differences (*p*<0.001) were found in the extent of inactivation in the two *L*. *pneumophila* associations. *L*. *pneumophila* associated with *Acanthamoeba* sp. 155 was much more sensitive to free chlorine than *L*. *pneumophila* associated with *A*. *castellanii* CCAP 1534/2. Moreover, although the effectiveness of chlorine treatment was higher at 2.5 mg L^-1^ in comparison to 1.2 mg L^-1^ for the *A*. *castellanii* CCAP 1534/2-associated *L*. *pneumophila*, the effectiveness of the *Acanthamoeba* sp. 155- associated *L*. *pneumophila* did not differ as much between these two concentrations. The inactivation kinetics of *L*. *pneumophila* associated with both, *A*. *castellanii* CCAP 1534/2 and *Acanthamoeba* sp. 155 fits a first-order model (Table 4). At 0.5 mg L^-1^, *L*. *pneumophila* reached a 4-log reduction after 5 min of treatment, but the FLA-associated *L*. *pneumophila* required 38 and 490 min to reach such a reduction when associated with *Acanthamoeba* sp. 155 and *A*. *castellanii* CCAP 1534/2, respectively. At 1.2 mg L^-1^, the time required for the cultivability of *L*. *pneumophila* associated with *A*. *castellanii* CCAP 1534/2 to be reduced 4 logs was 152 min, whereas for *Acanthamoeba* sp. 155-associated *L*. *pneumophila* it took only 17 min. Finally, at 2.5 mg L^-1^, *L*. *pneumophila* associated with *A*. *castellanii* CCAP 1534/2 and *L*. *pneumophila* associated with *Acanthamoeba* sp. 155 required 43 min and 23 min for such a reduction, respectively.

**Table 4:** Calculated time for a 4-log reduction of *L*. *pneumophila* sg. 1 env. associated with *A*.*castellanii* CCAP 1534/2 and *Acanthamoeba* sp. 155 after the exposure to different concentrations of free chlorine and temperatures. Inactivation kinetics adjusted to first-order models. R^2^ values showed the robustness of the models.

	Calculated time (min) to reduce 4 logs							
**Free chlorine**	**0.5 mg L** ^**-1**^	R^2^	**1.2 mg L** ^**-1**^	R^2^	**2.5 mg L** ^**-1**^	R^2^		
*L*. *pneumophila* sg.1 env (Axenic)	5	0.96	—	—	—	—		
*L*. *pneumophila* sg.1 env–*A*. *castellanii* CCAP 1534/2	490	0.85	152	0.76	43	0.79		
*L*. *pneumophila* sg.1 env—*Acanthamoeba* sp. 155	38	0.54	17	0.64	23	0.82		
**Temperature**	**50°C**	R^2^	**55°C**	R^2^	**60°C**	R^2^	**70°C**	R^2^
*L*. *pneumophila* sg.1 env (Axenic)	46	0.84	8	0.98	4	0.86	0.61	0.82
*L*. *pneumophila* sg.1 env–*A*. *castellanii* CCAP 1534/2	825	0.56	45	0.84	5	0.99	0.45	0.82
*L*. *pneumophila* sg.1 env—*Acanthamoeba* sp. 155	664	0.95	51	0.95	5	0.73	0.50	0.92

#### Thermal treatments

**Fig 7:**
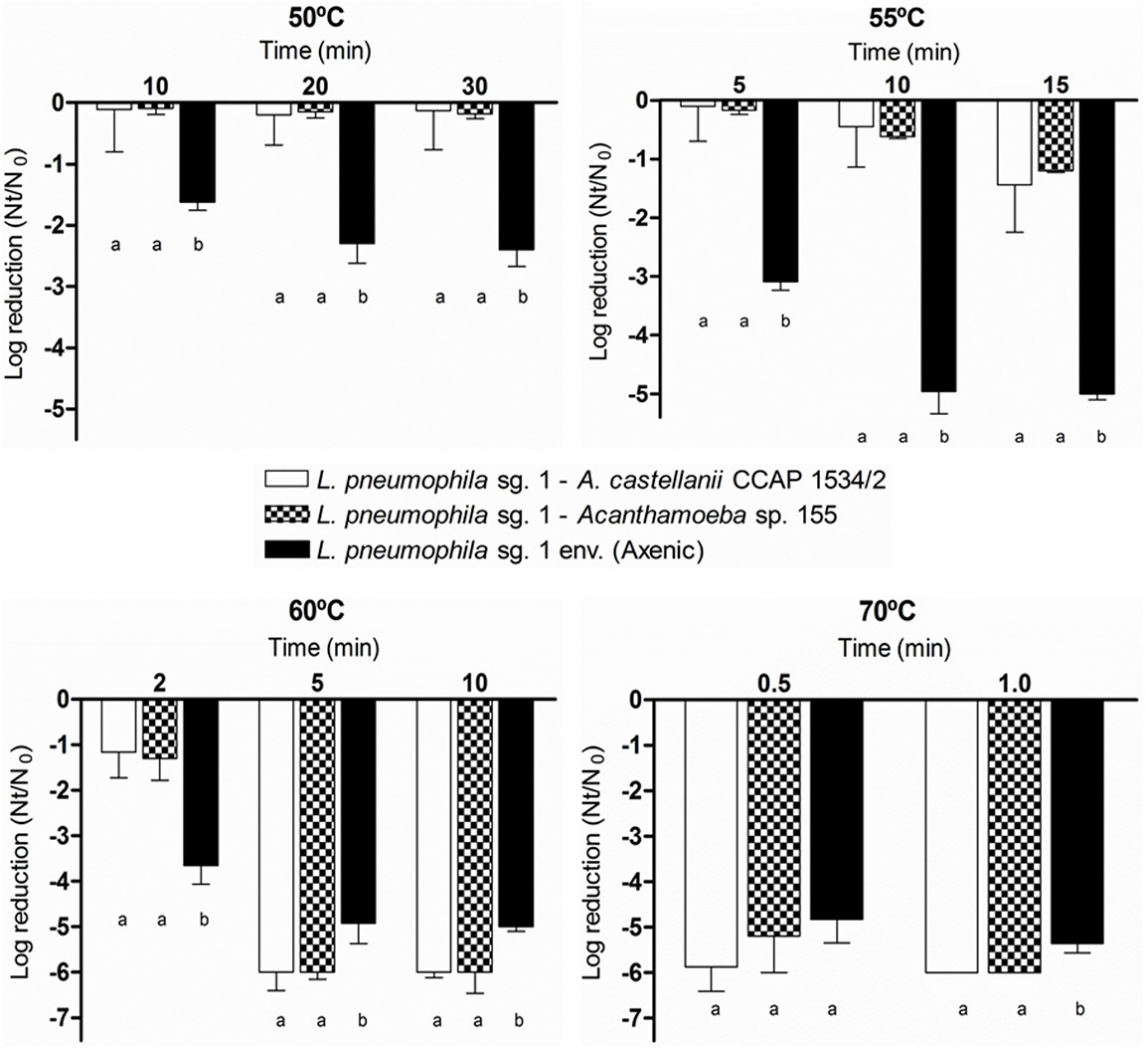
Effect of thermal treatments on the inactivation of *L*. *pneumophila* sg. 1 env. associated to two *Acanthamoeba* strains, *A*.*castellanii* CCAP 1534/2 and *Acanthamoeba* sp. 155 strains. Bacterial inactivation was determined using viable counts on BCYE medium. Data are presented as means ± SD (columns and error bars; n = 3). Statistical differences between means within each time point were represented assigning different letters to the bar plot. The same letter was assigned to bars with no significant differences between them. Statistical analyses were performed by ANOVA and pairwise Fisher’s LSD test (*p*<0.05).

The effect of thermal treatments at 50°C, 55°C, 60°C and 70°C was evaluated on *L*. *pneumophila* sg. 1 associated with *A*. *castellanii* CCAP 1534/2 and with *Acanthamoeba* sp. 155 (Fig 7). No significant differences were found (*p*>0.05) when comparing the inactivation pattern of the two associations in any of the thermal treatments applied. However, significant differences were found when the comparison was performed with axenic *L*. *pneumophila*. In that case, the effectiveness of the thermal treatment was significantly higher (p<0.001). Although the viability of *L*. *pneumophila* associated with *Acanthamoeba* trophozoites was reduced less than 0.5 log after 30 min at 50°C, the same strain in an axenic state was reduced more than 2 logs. At 55°C, the differences between the *L*. *pneumophila* associated experiments and the axenic *L*. *pneumophila* were the highest (p<0.001). Although the cultivability of *L*. *pneumophila* associated with *Acanthamoeba* trophozoites was reduced nearly 1.5 logs after 15 min, the cultivability of the same strain in an axenic state was 5 logs reduced. Remarkably, at 60°C and at 70°C, differences between the associated and axenic *L*. *pneumophila* were dramatically reduced (p<0.05) and no significant differences were observed between the three conditions tested.

The effect of thermal treatments on *L*. *pneumophila* associated with *Acanthamoeba* strains fits a first-order (straight line) model (Table 4). The time required for the cultivability of *L*. *pneumophila* to reach a 4-log reduction for the axenic *L*. *pneumophila* sg. 1 was 45 min at 50°C, 8 min at 55°C, 3 min at 60°C and 0.61 min at 70°C. When *L*. *pneumophila* associated with either *Acanthamoeba* strains, these times ranged from 664–825 min at 50°C, 45–50 min at 55°C, 4–5 min at 60°C and 0.45–0.50 min at 70°C.

## Discussion

The WHO drinking water quality guidelines recommend that the minimum target chlorine concentration at the point of delivery should be 0.2 mg L^-1^ in normal circumstances and 0.5 mg L^-1^ in high-risk circumstances [30,31]. Moreover, temperatures above 50°C are also recommended by the WHO [12] and in different national guidelines (e.g., the Spanish guidelines [2]) to avoid the colonization and regrowth of *Legionella* in the water systems. The aim of the present work was to model the effect of these disinfectants on the association between *L*. *pneumophila* and two *Acanthamoeba* strains and compare it with the models obtained for their free-living forms, which are usually the target in water disinfection treatments.

Five *Legionella* spp. strains were exposed to two free chlorine concentrations. At 0.2 and at 0.5 mg L^-1^ the strains fit a first-order model with the exception of the two *L*. *pneumophila* sg. 1 strains that showed a classic chlorine biphasic decay at 0.2 mg L^-1^ represented by an initial relatively rapid decay followed by a slower long-term decay rate. Specifically, *L*. *pneumophila* sg. 1 strains, particularly *L*. *pneumophila* sg. 1 ATCC 33152, were the most resistant to the conditions tested. The results obtained in the current study agreed with those reported by Kuchta et al. [32], who highlighted the fact that *Legionella* is more resistant to chlorine exposure than other bacteria such as coliform bacteria. Although Kuchta et al. [32] also reported significant differences in the inactivation pattern between *L*. *pneumophila* strains; they did not describe *L*. *pneumophila* sg. 1 strains as the most resistant.

The failure of disinfectants in controlling *Legionella* in drinking water systems has been attributed to the presence of protozoan hosts that act as a shield for pathogenic bacteria against disinfectants [18,33–36]. Because of that, two *Acanthamoeba* strains were exposed to 1.2 mg L^-1^ and 2.5 mg L^-1^ of free chlorine. Chlorine concentrations differed from the ones used in the case of *Legionella* due to the high resistance shown by the FLA in the previously conducted tests. Significant differences were observed between the inactivation patterns of the two *Acanthamoeba* stages, with the trophozoites being more sensitive than the cysts. Significant differences were found in the sensitivity of FLA strains. Inactivation models of *A*. *castellanii* CCAP 1534/2 trophozoites, which were the most sensitive at 1.2 and 2.5 mg L^-1^, fit a first-order model for both chlorine concentrations, whereas *Acanthamoeba* sp. 155 trophozoites fit a first-order model only at 2.5 mg L^-1^. On the other hand, *Acanthamoeba* cysts fit a biphasic decay model with a very slow decay rate for the free chlorine concentrations tested.

According to the literature, the effectiveness of free chlorine on the *Acanthamoeba* strains used in the current study was higher compared to those of *Giardia* [31], *Balamuthia* [37] or *Naegleria* [38]. Dupuy et al. [19] reported that a chlorine treatment between 2 and 3 mg L^-1^ inactivated at least 3 logs of all the *Acanthamoeba* strains they tested. However, as observed in the current study, the efficiency of the treatment applied varied depending on the target strain. Regarding *Acanthamoeba* cysts, several studies have reported its high chlorine resistence [18,39]. Considering the models reported here, we agree with Coulon et al. [40] that residual chlorine concentrations from 2 to 5 mg L^-1^ used to control the microbial biota in drinking water networks are ineffective against *Acanthamoeba* cysts.

Thermal treatments are usually applied in hot water systems of large buildings to control and prevent *Legionella* colonization [2,3,12]. In the current study, five *Legionella* spp. strains were exposed to different temperatures ranging from 50°C to 70°C in dialysis bags. The effectiveness of thermal treatments applied increased as the temperatures and exposure times increased, especially for temperatures higher than 55°C. However, significant differences were found when comparing the inactivation model for the different *Legionella* strains used. All thermal treatments applied were significantly more effective at reducing *L*. *longbeachae* ATCC 33462 compared to the *L*. *pneumophila* strains. *L*. *longbeachae* has been mainly isolated from potting soil [41], but some strains have also been found in water systems [42]. The adaptations that *L*. *longbeachae* undertook to survive in other ecosystems such as soil might be the cause of its lack of resistance to high temperatures. More tests should be performed to confirm that fact. Regarding the rest of the *L*. *pneumophila* strains, significant differences were observed between strains and serogroups. *L*. *pneumophila* sg. 1 ATCC was the most resistant at 50°C, whereas at 55°C, *L*. *pneumophila* sg. 7 was the most resistant. At higher temperatures (60°C, 65°C and 70°C), all *L*. *pneumophila* strains had a similar behavior.

*Legionella* strains used in the current study were more sensitive than the 19 members of the *Legionella*ceae family reported by Stout et al. [43]. Differences between these results may be explained by the use of dialysis bags, which ensure a fast and homogenous exposure to thermal treatments [24]. The effectiveness of thermal treatments was higher for *L*. *longbeachae* strains compared to *L*. *pneumophila* strains in both studies, and differences between *L*. *pneumophila* serogroups were also strain dependent. Since the current study was realized under controlled laboratory conditions it makes difficult to compare the results obtained with those in real water systems due to the large number of environmental factors involved [14,44]. However, although further research including these factors is needed, this study gives useful insights to better understand water disinfection dynamics.

Once the effect of free chlorine and temperature on individual *Legionella* spp. and *Acanthamoeba* spp. was characterized, inactivation models were assessed for two co-cultures between the environmental strain *L*. *pneumophila* sg. 1 and trophozoites of *A*. *castellanii* CCAP 1534/2 or *Acanthamoeba* sp. 155 exposed to the same disinfectant methods. The inactivation of *L*. *pneumophila* associated with *Acanthamoeba* strains fit a first-order model, as observed in the case of the free cells. However, the effectiveness of the chlorine treatments on the associated bacteria was reduced between 2.5 and 4 times at 0.5 mg L^-1^, the lowest chlorine concentration used. Although inactivation models were reported for the first time in the current study, the results obtained agreed with Dupuy et al. [19] and García et al. [11], who reported a higher resistance *L*. *pneumophila* to chlorine when it lived intracellularly within *Acanthamoeba* strains [11,19].

Regarding thermal treatments, inactivation models of *L*. *pneumophila* associated with *Acanthamoeba* trophozoites fit a first-order model, as did the free form. However, as in the case of chlorine, the effectiveness of the treatment compared to the free form was reduced, especially at the lowest temperatures. At 50°C, the bacterial resistance was increased between 14–18 fold, and at 55°C it was increased between 5 and 6 fold. The estimated models for the free *Legionella* strains showed a threshold of effectiveness at 55°C. However, *L*. *pneumophila* association with *Acanthamoeba* increased that threshold to 60°C. At that temperature, bacterial inactivation was similar between the two cell states. Thus, it seems that *Acanthamoeba* strains play a protective role for the bacteria at temperatures below 60°C, but at higher temperatures, its protection dramatically decreases. In fact, as observed in a previous study using the same exposure conditions [24], trophozoites of both *Acanthamoeba* strains were also significantly reduced at 60°C and even more sensitive than the bacteria in one case. Thus, it is likely that the *L*. *pneumophila* symbiont was reduced at a similar time as its trophozoite host. The results presented here agree with the only study on thermal resistance of the *Legionellae* symbiont, published by Storey et al. [18]. In that study, a 1-log increase in resistance of *L*. *pneumophila* and *L*. *erythra* to 50°C thermal treatments was reported for a symbiotic state.

Some authors have reported that stress conditions such as exposure to disinfectant including free chlorine or heat [45,46] can induce *Legionella* cells to enter a viable but non-cultivable (VBNC) state. Because of that, the effect of disinfection treatments on *Legionella* cells presented here could be even lower, as the viability quantification method used was based on the number of cultivable cells, which is still considered the gold standard today.

In summary, *Acanthamoeba* spp. is a natural inhabitant of drinking water systems that is able to survive the free chlorine concentration and temperatures used to ensure the microbial quality of the water system and to control and prevent *Legionella* colonization [current work, 36]. Based on the inactivation models reported in the current study, we described how *Acanthamoeba* survival would enhance the survival of associated *L*. *pneumophila* cells under those environmental conditions. This fact is especially important at critical points such as water outlets (taps) were water is stagnant and the disinfectant level, of either chlorine concentration or temperature, are even lower [14,47], promoting a *Legionella* regrowth and likely new legionellosis outbreaks. In conclusion, the results of this study can help to understand why some disinfection treatments are not efficient against *Legionella* and to design better treatments according to the disinfection models of amoeba-associated-*Legionella*.

## Conclusions

Legionellosis cases still occur from treated drinking water systems. The current work determines under controlled laboratory conditions how *L*. *pneumophila* resistance to common drinking water disinfection treatments is enhanced by its association with *Acanthamoeba* hosts. Inactivation models obtained showed that the increased resistance was remarkable for lower disinfectant exposures, 0.5 mg L^-1^ of free chlorine and temperatures of 50°C and 55°C. These conditions are commonly found in proximal areas of the water systems. On the other hand, free chlorine concentrations used in drinking water systems were ineffective against *Acanthamoeba* cysts. Therefore, amoebal survival and consequently the amoeba-associated *Legionella* should be considered when designing disinfection processes.
